# Primary eye care in Bhutan: achievements and challenges

**Published:** 2022-03-01

**Authors:** Nor Tshering Lepcha, Indra Prasad Sharma

**Affiliations:** 1 Ophthalmologist and Head: Gyalyum Kesang Choeden Wangchuck National Eye Centre, Jigme Dorji Wangchuck National Referral Hospital, Thimphu, Bhutan.; 2 Clinical Optometrist: Gyalyum Kesang Choeden Wangchuck National Eye Centre, Jigme Dorji Wangchuck National Referral Hospital, Thimphu, Bhutan.

**Long-term strategic action plans focused on strengthening primary eye care services have improved ophthalmic outcomes.**

Guided by the developmental philosophy of gross national happiness, Bhutan has made health care one of its top development priorities.^1^ In line with the principles of universal health coverage, primary health care is central to the country's public health policy. Bhutan's health services are predominantly financed and managed by the state; health financing is approximately 3.5% of the gross domestic product.

## Primary eye care centres

Bhutan established the state-funded primary eye care programme in 1987. It became a signatory to the VISION 2020 initiative in July 2000. Today 33 primary health centres across the country deliver primary eye care (PEC) services: among others, vision and refraction diagnosis, cataract screening, treatment of common eye conditions, and referral services. All PEC centres have slit lamps, refraction sets, and essential ophthalmic medications and are run by ophthalmic technicians recruited and trained by the Ministry of Health.

Despite the country's rugged terrain, improved roads and communications have improved the accessibility to PEC services. Outreach mobile operative eye camps cover the remotest population; starting with one camp in 1987, there were 20 in 2019. Due to the shortage of ophthalmic personnel, ophthalmic workers are mobilised by primary health centres to conduct eye camps. The national primary eye care programme facilitates the mobilisation of resources and funds for the outreach camps.

Annual school eye health programmes provide vision screening, refraction services, vitamin A supplementation, and treatment of minor eye ailments to all school children. The referral hospitals regularly organise outreach clinics in the primary health centres to provide ophthalmic speciality services.

In addition to the PEC centres, there are two centres for secondary eye care services and a dedicated, fully equipped national eye centre (inaugurated in 2019) for tertiary care.

## Disease control and coverage

The nationwide rapid assessment of avoidable blindness survey (RAAB 2018) estimated a 33% decrease in the prevalence of blindness in Bhutan between 2009 and 2018, thus achieving the objective of the WHO global eye health action plan 2014–2019.^2^^,^^3^ Between 2009 and 2018, there was a decrease in cataract blindness from 0.7% to 0.4%; and a substantial increase in cataract surgical coverage from 72.7% to 86.1%, combined with improved visual outcomes.^3^ Conjunctivitis was no more health morbidity in 2020 as it was in the past.^1^ Unoperated cataract and corneal opacities have also reduced in the last two decades.

## Service quality and efficacy

The promotion of manual small-incision cataract surgery and the universal use of intraocular lenses (IOL) have revolutionised cataract surgery; over 97.3% of people received an IOL implantation in 2018. Post-cataract surgical visual outcomes have improved: 67.3% of patients had good visual outcomes (VA>6/18) in 2018.^3^ The recruitment of eye specialists from neighbouring countries on a contract basis and the strengthening of secondary and tertiary centres have led to reduced ex-country referrals for advanced tertiary eye care needs.

## Human resources and planning

The training of allied ophthalmic personnel commenced in 1987, and a four-year ophthalmology residency programme was started in 2014. Starting with a lone ophthalmologist in 1987, Bhutan had 12 ophthalmologists, 9 optometrists, and 55 allied ophthalmic personnel in 2020. The aim is to achieve human resource self-sufficiency in the coming decade to staff all primary health centres with at least one ophthalmic assistant. To address the present shortage of ophthalmologists, optometrists and allied ophthalmic personnel could play a complementary role in the provision of non-surgical services.

**Figure 1:**
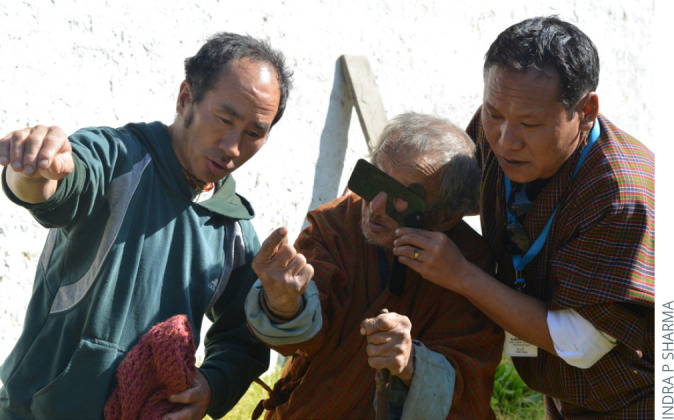
An elderly person gets screened for vision during the RAAB survey in 2018, Trongsa. **BHUTAN**

The use of data and evidence for planning PEC activities have helped improve eye care. The nationwide baseline (2009) and follow-up (2018) RAAB surveys (Figure 1) have been used to assess and plan PEC activities.^2^^,^^3^ In 2019, the school sight survey screened 175,000 school-aged children for refractive errors, and the results have aided in planning refractive services.^4^

## Challenges, changing trends, and the way forward

In Bhutan, the avoidable causes of visual impairment are still high (88.9%); 74.3% is attributable to cataract and refractive error. The volume of surgery (1,550 per million people) is lower than in other South-East Asian countries.^5^

With population growth and ageing, the incidence of refractive error and posterior segment diseases, including diabetic retinopathy, age-related macular degeneration, and glaucoma, is rising.^2^^,^^3^ To address this epidemiological transition, effective screening programmes must be embedded within the existing non-communicable diseases and primary health care frameworks. Paediatric eye health, including screening for retinopathy of prematurity, should be integrated into the broader child health initiatives. Long-term strategies for managing cataract and refractive error need to be developed. Population surveys and health systems research for effective PEC interventions are needed more than ever.

The ever-increasing healthcare costs, a declining share of external resources (owing to Bhutan's potential to graduate from the ‘least developed country’ status in 2023), and the COVID 19 pandemic are all undermining the sustainability of free health care.^6^ To ease the pressure on the already stretched health system and resources, we would suggest encouraging private sector participation and instituting some system of user fee model for eye health services.

## References

[1] Government of Bhutan. Annual Bulletin of Health. Ministry of Health. 2020. Available from: http://www.moh.gov.bt/wp-content/uploads/ict-files/2017/06/health-bulletin-Website_Final.pdf (accessed 18 April 2021).

[2] Lepcha NT Chettri CK Getshen K Rai BB Ramaswamy SB Saibaba S, et al. Rapid assessment of avoidable blindness in Bhutan. Ophthalmic Epidemiol. 2013; 20(4):212-9. 23841895 10.3109/09286586.2013.794902

[3] Lepcha NT Sharma IP Sapkota YD Das T Phuntsho T Tenzin N, et al. Changing trends of blindness, visual impairment and cataract surgery in Bhutan: 2009–2018. PLoS One. 2019; 14(5):e0216398. Available from: doi:10.1371/journal.pone.0216398 (accessed 15 April 2021). 31071127 10.1371/journal.pone.0216398 PMC6508732

[4] Sharma IP Lepcha NT Lhamo T Ellwein LB Pokharel GP Das T, et al. Visual impairment and refractive error in school children in Bhutan: the findings from the Bhutan school sight survey (BSSS 2019). PLoS One. 2020; 14;15(9):e0239117. Available from: doi: 10.1371/journal.pone.0239117 (accessed 15 April 2021). 10.1371/journal.pone.0239117 PMC7489552 32925975

[5] Das T Ackland P Correia M Hanutsaha P Mahipala P Nukella PB, et al. Is the 2015 eye care service delivery profile in Southeast Asia closer to universal eye health need! Int Ophthalmol. 2018; 38(2):469-80. 28255837 10.1007/s10792-017-0481-y

[6] UNICEF. UN agencies warn economic impact of COVID-19 and worsening inequalities will fuel malnutrition for billions in Asia and the Pacific. 2020. Available from: https://uni.cf/2SIKHRb (accessed 21 April 2021).

